# To the Editors of the Medical and Physical Journal

**Published:** 1800-02

**Authors:** John Bailey

**Affiliations:** Saint Thomas's Hospital


### *To the Editors of the Medical and Phyſical Journal.*

Gentlemen,

YOUR laſt Number contains an account of the exhibition of a remedy, which, of late, has been the cauſe of much diſputation in the medical world;—I mean, the *digitalis*; an article of the materia medica, about which, there has been a *ſtrange* diverſity of opinion. Some, with how much propriety I do not pretend to determine, deny its utility altogether; and with others, it has been the ſubject of much unmerited eulogy.

With reſpect to the former of theſe diſputants, I can poſitively aver, that I have witneſſed it to have the moſt benign and kindly influence over that diſeaſe, denominated hydrothorax. It will, as I conceive, be nugatory to take up time and room, which may be better occupied, in giving a relation of all theſe caſes; it may be ſufficient to ſay, that the remedy was employed under the direction and ſuperintendance of Mr. Hopkins, of Harwich, a gentleman well known in the county of Eſſex; and whoſe medical talents require no panegyric from my pen. In hæmorrhagy, I am informed, its efficacy ſtands almoſt unparalleled.

With regard to the latter, men who are prejudiced in favour of any particular remedy, ſee, or imagine ſo, effects produced by it, of which others diſcern no trait. The learned and very ingenious author (Dr. Drake) of the paper before alluded to, attributes wonderful ſalutary effects to the digitalis, as adminiſtered *by him* in phthiſis pulmonalis tuberculoſa; he even goes ſo far as to ſay, that it may almoſt be conſidered as a ſpecific. I do, with the utmoſt deference, bow down to the Doctor's ſuperior ability; I know full well his accuracy and preciſion in marking the ſymptoms of diſeaſe, and his judgment in diſcerning the effects of remedies; but I cannot help obſerving, that I think him here too ſanguine.

Some time ago, I was induced to give trial to this remedy in a caſe of tubercular conſumption, about which there was no manner of doubt; I entertained hopes, not a little ardent, of its having beneficial effects; but was ſurprized to find, that it produced no one that ſeemed in the leaſt to arreſt the progreſs of the diſeaſe, or even to mitigate the ſufferings of the patient: in fact, it tended rather to increaſe them; the failure of it I knew could not to be attributed to the irregularity of the preparation, becauſe it was agreeable to that which the doctor recommends. During my avocations at this hoſpital, many trials have been given it, and the reſults have fallen infinitely

ſhort

ſhort of what was expected; in but a few inſtances could patients be ſaid to receive even a palliation of ſymptoms. Theſe trials were made under the direction of gentlemen, who uſed every effort to avail themſelves of its utility. I could adduce many inſtances of the failure of this remedy in tubercular conſumption; in fact, I have the authority of men eminent in their profeſſion,—men who ſtand at the head of medical ſcience, for declaring, that digitalis never yet has cured a perſon labouring under this complaint. It is moſt certainly true, that digitalis, when judiciouſly adminiſtered in the incipient ſtage of phthiſis, will, like any other ſedative, retard the progreſs of the diſeaſe; but who truſts to this alone? Is not every exciting cauſe removed? An abſtinence from all ſtimuli; the viciſſitudes of weather guarded againſt, &c.? If the patient ſhould be ſnatched from the impending danger, how can it be ſaid that digitalis has effected the cure? Seeing, then, that there muſt be an aſſemblage of remedies, the effects of all which are, perhaps, fortuitous, to reſtore the patient to his priſtine vigour,—can it with propriety be adjuged to any one in particular?

The modus operandi of digitalis, is not at all difficult to be explained; its effect on the human body, is that of a *direct* ſedative; like opium, it reſtrains the action of heart and arteries, through the medium of the ſtomach; i. e. by the harmony that ſubſiſts between thoſe organs; conſequently the blood is propelled with leſs energy to the pulmonic ſyſtem. Inflammation, if it has begun, is removed; and thus the diſeaſe may for a time be ſuſpended. Beyond this ſedative effect, digitalis avails us nothing.

I am, Gentlemen,

Your's, &c.

JOHN BAILEY.

*Saint Thomas's Hoſpital,*

*Dec.* 16, 1799.

---

### *To the Editors of the Medical and Phyſical Journal.*

Gentlemen,

